# Description of *Neoperla mindoroensis* sp. nov., the first record of a stonefly from Mindoro, Philippines (Plecoptera, Perlidae), and identification of its life stages using COI barcodes

**DOI:** 10.3897/zookeys.954.53746

**Published:** 2020-07-29

**Authors:** Arthien Lovell Pelingen, Hendrik Freitag

**Affiliations:** 1 Department of Biology, School of Science and Engineering, Ateneo de Manila University, Quezon City, Philippines Ateneo de Manila University Quezon Philippines

**Keywords:** DNA barcode, integrative taxonomy, Mt Hinundungan key biodiversity area, new species

## Abstract

The new stonefly species, *Neoperla mindoroensis***sp. nov.** (Perlidae), from Mindoro island is described. The new species is assigned to the *N. recta* species complex of the *N. montivaga* group on account of its obvious T7 and T8 with pointed processes and the presence of basolateral lobes in the everted aedeagal sac. The male adult is distinguishable by its aedeagus with a slightly raised mediodorsal lobe, fully covered with fine spinules, while the female adult has comparably small eggs (240 × 220 μm) with a punctate, chorionic surface with punctae arranged in polygonal FCIs. The life stages and sexes were assigned using COI mtDNA barcodes (2.2% maximum intraspecific genetic distance), which were compared with available barcodes of congeners, which had interspecific genetic distances varying by at least 23.5%. Biogeographic aspects, ecological habitat requirements, and suitability as potential bioindicator of the species are also briefly discussed.

## Introduction

Plecoptera (stoneflies) is a basal, aquatic order of Neopteran insects known for their intolerance to organic pollution (Fochetti and Tierno de Figueroa 2008). Their presence and abundance are important in rapid assessments of water quality in freshwater ecosystems, especially rivers and streams, as the order occurs worldwide, except in Antarctica (DeWalt and Ower 2019).

One of the most diverse stonefly genera in the Oriental Realm and Southeast Asia is *Neoperla* Needham, 1905. In fact, in the Philippines alone, there are already 23 recorded *Neoperla* species even if these are only known from few major islands (Jewett 1958; Kawai 1969; Sivec 1984; Zwick 1986; Sivec and Stark 2011; Dela Cruz et al. 2018) and have not been comprehensively sampled yet. Interestingly, some closely related species occur syntopically (Sivec 1984).

Usually, only the male adult stages of stoneflies were formally described from the Philippines (Zwick 1982, 1986; Sivec 1984; Sivec and Stark 2011). Conspecificity with female adults and nymphs were ambiguous due to a lack of material or suitable matching tools, which made it difficult to use them as bioindicators in freshwater assessments. In recent decades, DNA barcoding has increasingly been used in associating life stages of aquatic insects with their adults (Freitag 2013; Dela Cruz et al. 2016; Garces et al. 2018). For the first time, a new set of modified primers is applied here to Philippine Plecoptera.

This study focuses in the Baroc River Catchment, which is in the Key Biodiversity Area “69 Hinunduang Mt.” (*sensu*Ong et al. 2002) on Mindoro island. Here, several interesting aquatic insects have been discovered during the comprehensive assessment of the Ateneo Biodiversity Laboratory (Freitag 2013; Mey and Freitag 2013; Komarek and Freitag 2014; Vidal et al. 2017; Garces et al. 2018). This study also aims to address the lack of taxonomic studies of stoneflies from this biogeographically interesting island, where no stonefly records have been known until now, and specifically to describe one new *Neoperla* species based on male and female adults along with the associated nymphs identified by COI mtDNA barcodes.

## Methods

As part of a freshwater biodiversity assessment project, the stonefly fauna of the Baroc River Catchment, Roxas, Oriental Mindoro, was sampled in 2018–2019. Nymphs were collected by manual collection from rock surfaces, submerged wood, and trapped leaf packs in riffle sections of the Baroc River and its tributaries, while the adults were collected by the use of black-light traps and emergence traps as described by Freitag (2004). The collections were preserved in 95% ethanol and stored at –20 °C at the Biodiversity Laboratory, Ateneo de Manila University until scientific treatment. The following codes were used to identify the sampling sites (all belong to the Baroc River System and are within the area of Barangay San Vicente, Roxas Municipality, Oriental Mindoro, the Philippines); maps indicating collections sites and notes are provided by Mey and Freitag (2013: 301) and Vidal et al. (2017: 2–5):

HBT: Quirao Buhay Creek tributary Tagugoy Creek, disturbed secondary forest; ca 12°36'30"N, 121°22'38"E, 200 m a.s.l.

HOC: Hinundungan River tributary Quianao Creek, secondary forest; ca 12°35'20"N, 121°21'40"E, 280 m a.s.l.

HR3: upper Hinundungan River, secondary forest; ca 12°35'10"N, 121°21'36"E, 280 m a.s.l.

TBC: Taugad Daka River tributary Batuwayang Creek, secondary forest; ca 12°38'09"N, 121°19'45"E, 490 m a.s.l.

TDR1: Taugad Daka River near Sitio Taugad Diit, rural extensive farmland and secondary vegetation; ca 12°37'33"N, 121°21'18"E, 180 m a.s.l.

TDR3: upper Taugad Daka River, secondary forest; ca 12°38'05"N, 121°19'33"E, 530 m a.s.l.

THC: Taugad River tributary Hiyong Creek, rural extensive farmland and secondary vegetation; ca 12°37'27"N, 121°22'48"E, 147 m a.s.l.

TIR: Taugad Diit River near Sitio Taugad Diit, rural extensive farmland and secondary vegetation; ca 12°37'32"N, 121°22'17"E, 180 m a.s.l.

TR2: Taugad River downstream Sitio Taugad Diit, secondary vegetation; ca 12°37'18"N, 121°22'58"E, 140 m a.s.l.

The external morphology of the specimens was studied under a Leica EZ4 stereomicroscope. The cold maceration technique (Zwick 1982) was employed to properly observe the aedeagus. The aedeagus, aedeagal sac, female inner genitalia, and the nymphal mouthparts were examined as wet mounts on microscopic slides under an Olympus CX21 compound microscope. Digital imaging of dissected parts was done using these microscopes with a DinoEye Eyepiece camera, then stacked using CombineZP software (Hadley 2010). The female inner genitalia were drawn in Adobe Illustrator 2020. The images of habitus and male terminalia were produced using a Canon EOS 650D and a Canon EOS 6D, respectively, with macro lens and a stack rack operated by Helicon Remote, and then stacked using Helicon Focus. Stacked images were enhanced with Adobe Lightroom and Adobe Photoshop 2020. Preparation of eggs was done following the procedure of Sivec et al. (1988) and examined and photographed using a Hitachi TM-1000 Table Top Scanning Electron Microscope (SEM) at the Materials Physics Laboratory, Ateneo de Manila University. Terminologies follow Murányi et al. (2015).

All type material is stored in alcohol and has been deposited at the Museum of Natural History of the National Museum of the Philippines, Manila, Philippines (**NMP**); Biodiversity Laboratory, Ateneo de Manila University, Quezon City, Philippines (**AdMU**); Collection Arthien Pelingen, Philippines (**CAP**), currently deposited in AdMU; Museum für Naturkunde Berlin, Germany (**ZMB**).

DNA was extracted from the legs using Qiagen DNeasy kit (Qiagen, Hilden, Germany) following the protocol for animal tissues (Qiagen 2002). The 5'-end of the cytochrome c oxidase subunit I (COI) region was then amplified using the primers LCO1490_mod (5'-TTTCAACAAACCATAAGGATATTGG-3') and HCO2198_mod (5'-TAAACTTCAGGATGRCCAAAAAATCA-3') (Garces et al. 2018). In a 25 µl Polymerase Chain Reaction (PCR) mix, it includes 17.8 µl ddH_2_O, 2.5 µl 10× buffer, 1 µl Mg (25 mM), 0.5 µl dNTP mix (10 mM), 0.5 µl of each primer (10 mM), 0.2 µl Taq Polymerase (NEB), and 2 µl template DNA of unknown concentration. The PCR program was set as follows: 180 s at 94 °C; 30 s at 94 °C, 30 s at 47 °C, 60 s at 72 °C (× 35 cycles); 300 s at 72 °C. The amplification success was then checked in a 1.5% agarose gel using gel electrophoresis. The successfully amplified PCR products were sent to MACROGEN for cleaning and sequencing. The forward and reverse sequences were then manually traced and aligned (CLUSTALW) using BIOEDIT v. 7.2.5 (Hall 1999) along with the corresponding partial COI sequences of *Neoperla clymene* (Newman, 1839) and *Neoperla obliqua* Banks, 1913 retrieved from GenBank as seen in Table 1 (Pilgrim et al. 2011; Dela Cruz et al. 2018). A statistical parsimony analysis was conducted with TCS (Clement et al. 2002), and the haplotype network was visualized using POPART v. 1.7 (Leigh and Bryant 2015) and edited in Adobe Illustrator 2020.

The pairwise genetic distance analysis was performed in MEGA 7 (Kumar et al. 2016) using Kimura-2-parameter (K2P) model with bootstrap method in 1000 replicates.

**Table 1.** GenBank accession numbers of DNA sequences, geographical origins, collection sites, and sample references of specimens. External data are indicated by superscript numbers: ^1^Dela Cruz et al. 2018; ^2^Pilgrim et al. 2011.

Species	Locality	Stage	Voucher	Genbank accession number	GenSeq nomenclature
*Neoperla mindoroensis* sp. nov.	Mindoro	nymph	PL21	MT547994	genseq-2 COI
	Mindoro	♀ adult	PL22	MT547995	genseq-2 COI
	Mindoro	♂ adult	PL50	MT547996	genseq-1 COI
*Neoperla obliqua*	Mindanao	♂ adult		KT307712 ^1^	
	Mindanao	♀ adult		KT307713 ^1^	
*Neoperla clymene*	USA			JN200655 ^2^	

## Taxonomy

### Neoperla mindoroensis sp. nov.

Taxon classificationAnimaliaPlecopteraPerlidae

868D7173-E96F-5084-BB0E-B2C9005B7963

http://zoobank.org/22178DC3-B257-48B3-BDED-3193FFDF7F4F

#### Type locality.

Philippines • Oriental Mindoro, Municipality of Roxas, Barangay San Vicente: Quirao Buhay Creek tributary Tagugoy Creek; secondary forest, ca 12°36'30"N, 121°22'38"E, ca 200 m asl.

#### Material.

***Holotype***: 1 ♂ adult (NMP), labelled “PHIL: Or[iental]. Mindoro, Roxas, Brgy. San Vicente, Quirao \ Buhay tributary, Tagugoy Creek; secondary forest; \ 12°36'30"N, 121°22'38"E 200 m a.s.l.; leg. AL Pelingen, \ C Pangantihon, H Freitag 05 Feb. 2018 (HBT)L”, preserved in a cryovial with 95% ethanol, right hindleg and all left legs missing as used for DNA extraction (PL50), both cerci partially broken, tips of wings partially broken, dissected aedeagus stored inside the same vial. ***Paratypes***: Philippines • 1 ♂ adult; HBT E; 12 Aug.–21 Sept. 2018; leg. Freitag & Pangantihon; NMP; left midleg and hindleg missing, both cerci partially broken, dissected aedeagus stored inside the same vial • 1 ♂ adult; HR3 E; 15 Jan.–17 Feb. 2019; leg. Pangantihon; ZMB; both cerci partially broken, dissected aedeagus stored inside the same vial • 1 ♀ adult; TDR1 L; 08 May 2018; leg. Freitag and Pangantihon; NMP; right legs used for DNA extraction (PL22), both cerci partially broken, eggs used for SEM • 1 ♂ nymph; TDR1f; 22 Sept. 2019; submerged wood in run; leg. Freitag and Pangantihon; AdMU; dissected mouth parts stored in the same vial • 1 ♀ nymph; TDR3f; 08 Feb. 2018; submerged wood in run; leg. Freitag; NMP; left midleg and hindleg used for DNA extraction (PL21); left foreleg broken but stored in the same vial, both cerci partially broken • 1 ♂ adult; TDR3/TBC L; 08 May 2018; leg. Freitag & Pangantihon; CAP-AdMU; right and left midlegs broken but stored inside the same vial, both cerci partially broken, dissected aedeagus stored inside the same vial • 1 ♀ adult; TDR3/TBC L; 08 May 2018; leg. Freitag & Pangantihon; AdMU; left hindleg missing, both cerci partially broken, right forewing broken but stored in the same vial • 1 ♀ adult; THC E; 19 Nov.–02 Dec. 2018; leg. Freitag; ZMB; both cerci broken • 1 ♂ adult; TIR E; 24 Jan.–16 Feb. 2018; leg. Pangantihon; ZMB; right cercus partially broken, dissected aedeagus stored inside the same vial • 1 ♂ adult; TIR E; 24 Jan.–16 Feb. 2018; leg. Freitag; AdMU; left hindleg missing, both cerci partially broken, tips of wings partially broken, dissected aedeagus stored inside the same vial • 1 ♀ adult; TR2 L; 11 Aug. 2019; leg. Freitag; ZMB; both cerci partially broken • 1 ♀ adult; TR2 L; 11 Aug. 2019; leg. Freitag & Pangantihon; NMP; left midleg missing, both cerci partially broken • 1 ♂ adult; TR2 E; 22 Dec. 2018–15 Jan. 2019; leg. Freitag & Pangantihon; AdMU; right midleg broken but stored in the same vial, both cerci partially broken, wings damaged, dissected aedeagus stored inside the same vial. ***Other Material***: Philippines • 1 ♂ larva; HOCg; 16 Jan. 2019; rock surface in riffle, leg. Freitag; AdMU; right hindleg missing, both cerci partially broken • 1 ♀ larva; TIRd; 22 Sept. 2019; leaf pack in riffle; leg. Freitag and Pangantihon; AdMU; both cerci partially broken • 1 ♂ larva; TIRd; 22 Sept. 2019; leaf pack in riffle; leg. Freitag and Pangantihon; CAP-AdMU; segments IX and X including cerci missing • 4 ♀ adults; TR2 E; 19 Nov.–02 Dec. 2018 leg. Freitag; AdMU; some legs missing and all cerci partially broken • 3 ♀ adults; TR2 E; 22 Dec. 2018–15 Jan. 2019; leg. Freitag & Pangantihon; AdMU; some legs missing and all cerci partially broken.

#### Description.

**Figure 1.**
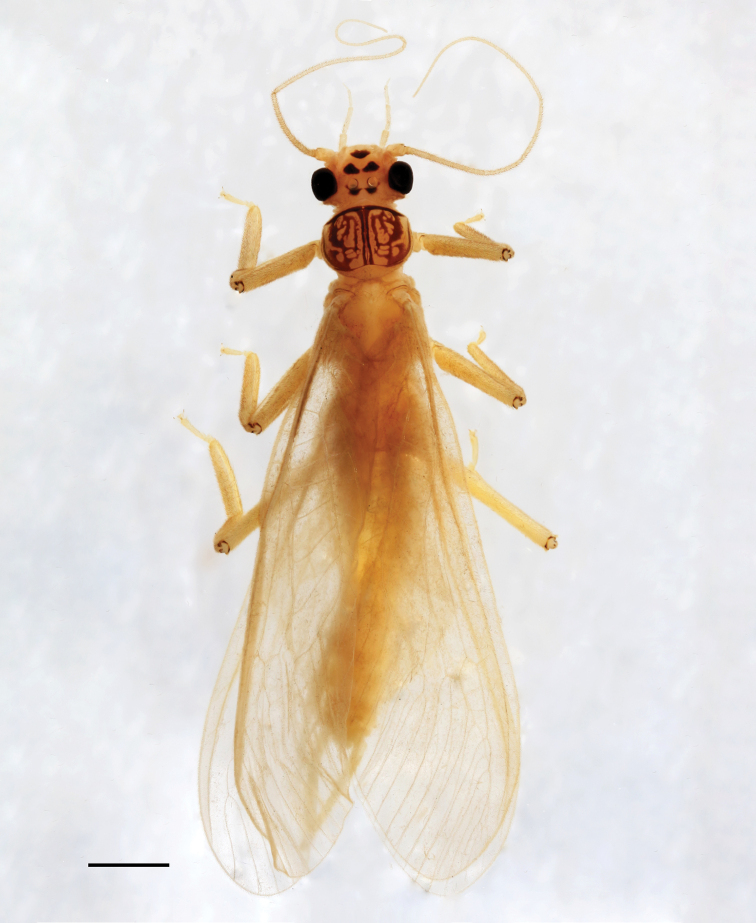
Paratype male adult habitus of *Neoperla mindoroensis* sp. nov. Scale bar: 2.00 mm.

***Imago***: Medium-sized species (Fig. 1). Forewing length of holotype male: 14 mm, paratype males: 14–18 mm, paratype females: 16–18 mm. General color pale with dark patterns. Ocelli relatively of the same size in both male and female; distance between ocelli more than its diameter in male, less than its diameter in female. Head predominantly pale; dark mottling present posterior of ocelli; with two triangular, dark-brown patches anterior of ocelli and another patch near anterior of head delimiting a pale but distinct M-line. Antenna and palpi slightly darker than head. Pronotum trapezoidal, narrower than head with eyes; anterior edges slightly angled; ground color brown with distinct yellow rugosities and with medial, longitudinal, brown stripe and dark, transverse anterior and posterior lines. Meso- and metanotum pale brownish. Legs yellow; tibia darker than the rest of the legs. Wings hyaline, nearly transparent; veins brown.

**Figure 2.**
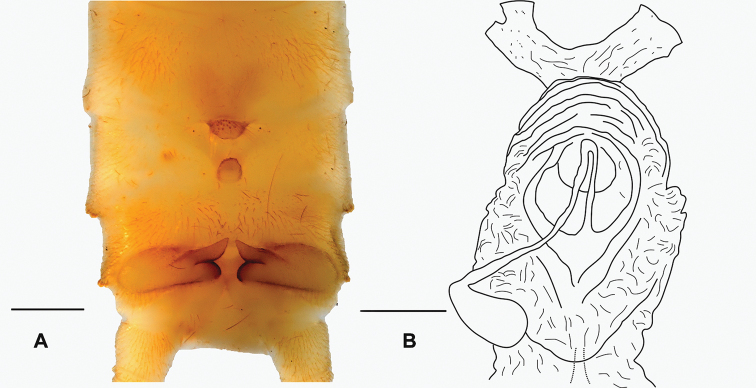
**A** male terminalia of *Neoperla mindoroensis* sp. nov. **B** female inner genitalia. Scale bars: 0.5 mm (**A**); 0.1 mm (**B**).

***Male terminalia*** (Fig. 2A): Sterna and terga 2–6 simple. Posterior process of tergum 7 with large, median hump associated with sparsely arranged and long setae, and sensilla basiconica on its hump. Tergum 8 with distinct medial process, strongly curved anteriorly like a hook, bearing dense sensilla basiconica. Tergum 9 simple, with irregular, sparse setation throughout. Posterolateral margin of segments 7–9 with rows of moderately densely arranged, stout, brown setae. Hemitergal lobe covered with fine setae. Hemitergal processes short, not raised in lateral view, slightly bent anteriad subparallel to midline.

***Female terminalia*.** Terga and sterna simple; subgenital plate with slightly bilobed posterior edge of S8, half as wide as segment’s width; inner genitalia (Fig. 2B) unsclerotized and transparent, with distinct lamellae attached to the receptacle stalk; concentric and lateral folds discernable around and apically of the seminal receptacle’s attachment, respectively.

**Figure 3.**
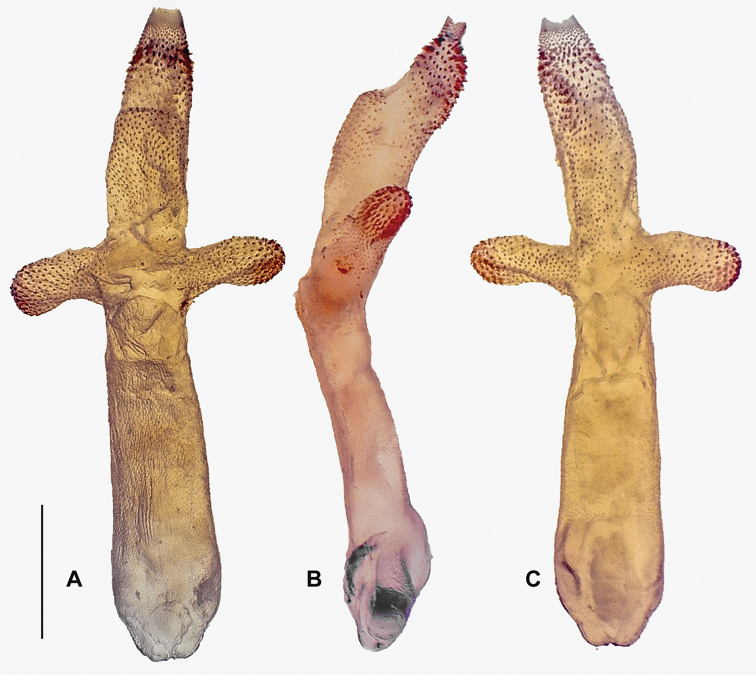
Aedeagus of *Neoperla mindoroensis* sp. nov. **A** dorsal **B** lateral **C** ventral. Scale bar: 0.1 mm.

***Aedeagus*** (Fig. 3A–C): Aedeagal tube slightly bulky, with dorsobasal and short elongate, ventrobasal sclerites; basoventral surface of tube with hump. Dorsal surface of entire aedeagal tube with wrinkles, but entirely without any spines. Everted aedeagal sac bent slightly ventrad, shorter than aedeagal tube; basolateral lobes with strong apical spines and smaller spinules basally, posterobasal area almost glabrous; mediodorsal lobe slightly raised, fully covered with fine spinules; subapical portion with strong spines, basad increasingly with fine spinules on ventral surface; lateral and dorsal areas around the mediodorsal lobe almost glabrous.

**Figure 4.**
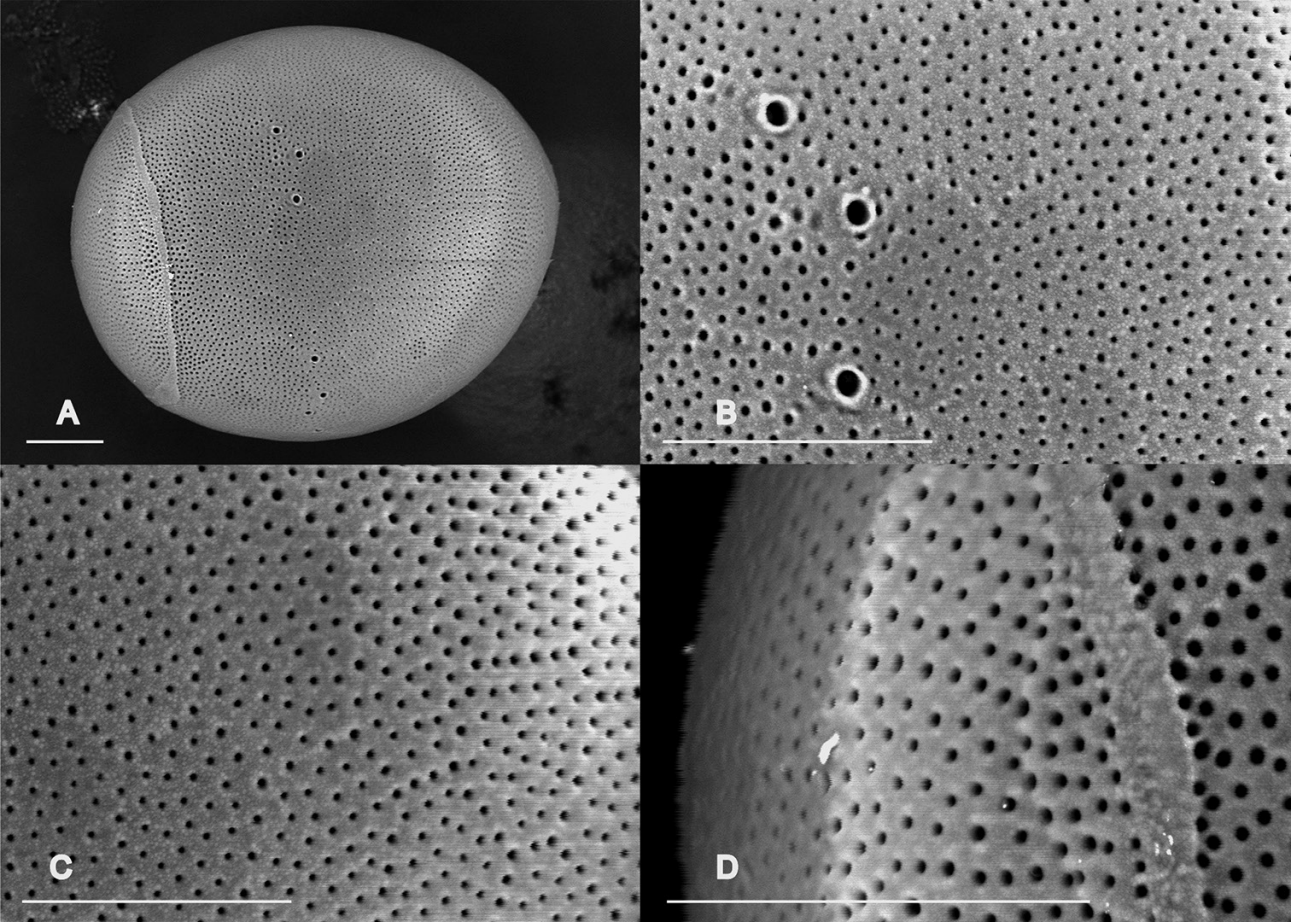
SEM micrographs of the egg of *Neoperla mindoroensis* sp. nov. **A** full egg **B** micropyles **C** chorion surface **D** hatching line. Scale bars: 20 µm.

***Egg***: Color dark brown, oval, nearly spherical, length ca 240 μm, width ca 220 μm, hatching line visible. Chorionic surface regularly punctate throughout, with punctae arranged in polygonal FCIs. Micropyles without any grouped rims near the hatching line (Fig. 4A–D).

**Figure 5.**
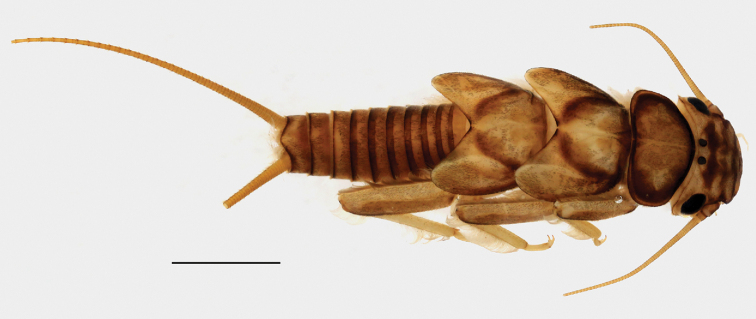
Female larval habitus of *Neoperla mindoroensis* sp. nov. Scale bar: 4.0 mm.

***Nymph***: General color pale brown, abdomen darker brown (Fig. 5, larva with identical pattern). Venter pale brown. Female total length 16–18 mm. Male total length 12–13 mm.

**Figure 6.**
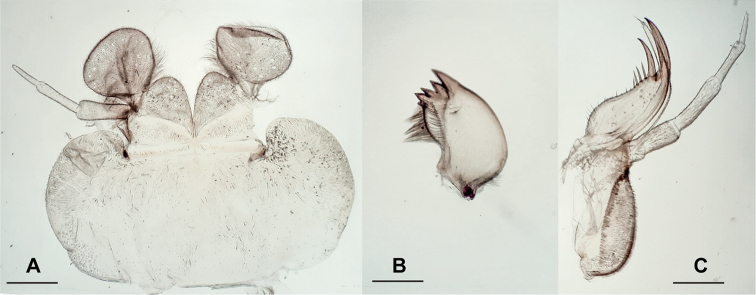
Nymphal mouthparts of *Neoperla mindoroensis* sp. nov. **A** labium, ventral **B** left mandible **C** left maxilla Scale bars: 0.1 mm.

***Head*.** Pale, predominantly brownish, slightly wider than pronotum, margins with black outline. M-line pale and tentorial callosities indistinct; stem of ecdysial suture forms a white line which opens in a white spot in the middle of the dark markings anterior of occipital area. Frons simple, with bands of mottlings. Distance in between ocelli slightly greater than their diameter. Antennae longer than combined pro-and mesothorax, yellow. Labium, labial palp, paraglossae, glossae (Fig. 6A), mandible (Fig. 6B), maxilla (Fig. 6C) family-typical. Mandible (Fig. 6B) with deeply curved molar and five uneven incisors. Maxilla (Fig. 6C): lacinia scythe blade-like with broad basal half, subapical tooth a third shorter of the apical tooth, four large setae and few smaller setae in the marginal fringe, galea almost as long as lacinia with thin apical seta.

***Thorax*.** Pronotum with yellow middorsal stripe and dark margins. Meso- and meta-notum with yellow mid-dorsal stripe; dark bands extending from mid-length to anterior corners lining borders of wingpads; additional dark markings evident on all thoracic segments. Legs yellow, proportion 1.0:1.3:1.5; proleg: 6.0–6.5 mm, midleg: 7.0–8.0 mm, hindleg: 9.0–10.0 mm long; posterior of all legs entirely lined with very fine, dense setae; setae ca 0.5 mm long. Thoracic gills very dense, length up to 1.0 mm.

***Abdomen*.** Posterior margins of abdominal segments with distinct dark bands. Terga sparsely covered with short, very fine, dark hairs; terga II–X with thin and sharp intercalary setae. Cerci yellow, about half as long as body; cercal hairs short and blunt. Segment X with one pair of anal gills, of approximately 20 filaments in each cluster, ca 0.5 mm long.

#### Differential diagnosis.

*Neoperla mindoroensis* sp. nov. imagines are similar to *Neoperla nishidai* Sivec, 1984 from Greater Palawan in having pointed processes in terga 7 and 8 and in the two large, finger-shaped basolateral lobes at the aedeagal sac. However, *N. nishidai* has smaller T8 process, and its basolateral lobes and the aedeagal sac are dorsally covered by spines and bare ventrally, while in *N. mindoroensis* sp. nov. the basolateral lobes are densely armed with spinules, and possess a fully spinulose, slightly raised mediodorsal lobe on the sac. The aedeagal sac of *N. nishidai* was also described as strongly bent ventrally, while *N. mindoroensis* sp. nov. is only slightly bent ventrally. Additionally, the egg of *N. mindoroensis* sp. nov. is significantly smaller (240 × 220 μm) and has less pronounced FCIs than that of the supposedly conspecific female of *N. nishidai* (340 × 300 μm) (Sivec 1984). *Neoperla* PA-9 (Sivec and Stark 2011: 272, 273), which was claimed to be the putative true female of *N. nishidai*, also has larger eggs (271 × 256 μm) and an entirely different morphology from *N. mindoroensis* sp. nov. In addition, *Neoperla* PA-9 egg has a thin and obscure opercular line, but bearing a series of small, raised spine-like processes, while *N. mindoroensis* sp. nov. does not have any spine-like structure. The aedeagus of *N. palawan* Sivec & Stark, 2011 also resembles that of *N. mindoroensis* sp. nov., but its basolateral lobes are distinctly smaller, rounded, and not elongate, with a low, rounded medioventral lobe. In addition, it does not have a prominent T8 process on the dorsal abdomen. From all other male adult Philippine *Neoperla*, the new species can easily be distinguished externally by the distinct, complex pattern in its pronotum, structure of its hemitergites, and its genitalia, as described above. The female adult bears the same pronotum pattern.

#### Etymology.

The toponym refers to the Philippine island of Mindoro, where the type locality is situated.

#### Distribution.

This species is known so far only from the Baroc River Catchment, Roxas, Oriental Mindoro, Philippines.

#### Ecology.

**Figure 7.**
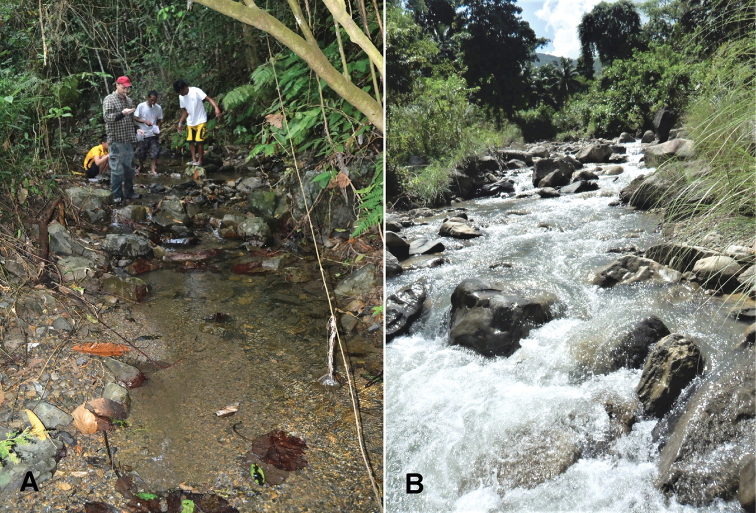
Type locality and an additional sampling site of *Neoperla mindoroensis* sp. nov. **A**HBT**B**TIR (Photos by Mr. Clister Pangantihon).

In the Baroc River Catchment, the specimens were found in altitudes of 140–530 m a.s.l. from Hinundungan River and Tauga River tributaries (Fig. 7). These collection sites were surrounded by either secondary forest or rural extensive farmland, if not secondary vegetation. Along these small to medium-sized (0.4–12 m wide) streams, the nymphs were found on submerged leaf packs, woods, and rock surfaces in riffle sections. In these microhabitats, the following physico-chemical variables were measured or estimated: water current 0.01–0.93 m/s, water temperature 21.5–26.8 °C, pH 7.5–8.5, dissolved oxygen 6.7–8.75 mg/L (mostly, but not always near 100% saturation), biochemical oxygen demand (BOD_5_) 0.1–1.18 mg/L. The maximum values for dissolved nutrients were 0.5 mg/L phosphate and 1.0 mg/L nitrate.

### Updated checklist of *Neoperla* Needham, 1905 from the Philippines

*N. agtouganon* Sivec & Stark, 2011 (Mindanao)

*N. agusani* Sivec, 1984 (Mindanao)

*N. andreas* Sivec & Stark, 2011 (Palawan)

*N. atripennis* Banks, 1924 (Leyte, Mindanao)

*N. connectens* Zwick, 1986 (Borneo, Mindanao)

*N. dentata* Sivec, 1984 (Borneo, Busuanga, Palawan)

*N. flinti* Sivec, 1984 (Luzon, Mindanao)

*N. hermosa* Banks, 1924 (Mindanao)

*N. jewetti* Sivec, 1984 (Luzon)

*N. mindoroensis* sp. nov. (Mindoro)

*N. nigra* Sivec, 1984 (Luzon)

*N. nishidai* Sivec, 1984 (Busuanga, Palawan)

*N. obliqua* Banks, 1913 (Luzon, Mindanao)

*N. oculata* Banks, 1924 (Biliran, Leyte, Luzon, Mindanao)

*N. palawan* Sivec & Stark, 2011 (Palawan)

*N. pallescens* Banks, 1924 (Mindanao)

*N. pallicornis* Banks, 1937 (Leyte, Luzon, Samar)

*N. philippina* Sivec, 1984 (Busuanga)

*N. pseudorecta* Sivec, 1984 (Busuanga, Cebu, Luzon, Negros, Palawan)

*N. recta* Banks, 1913 (Luzon, Negros, Mindanao)

*N. sabang* Sivec & Stark, 2011 (Palawan)

*N. salakot* Sivec & Stark, 2011 (Palawan)

*N. wagneri* Sivec, 1984 (Mindanao)

*N. zwicki* Sivec, 1984 (Luzon, Mindanao, Samar)

## Discussion

Among the four stonefly genera which are known from the Philippines, *Neoperla* is the best documented genus in the country, with currently 24 species as listed above. For the first time, an integrative taxonomic approach was applied to describe a new Philippine *Neoperla* species using a newly designed primer. The same primer has proved to be efficient in generating DNA barcodes of Ephemeroptera species (Garces et al. 2018, 2020). In a preliminary analysis of a comprehensive assessment of Swiss stoneflies (Gattolliat et al. 2016), one of the factors pointed out is the need to explore more specific primers other than the standard COI primers (Folmer et al. 1994) for better output. In this study, the barcodes have been instrumental to associate the different stages of the new species and differentiate them from congeners of which mtDNA sequences are available. As a result, the female adult and the nymph were also properly described.

In biodiversity surveys of aquatic insects and ecological assessments of rivers (e.g. Junqueira et al. 2010), the nymphs, which are usually difficult to be identified to species level, are commonly collected and not the adult forms. Among all Oriental *Neoperla* recorded, the only nymphal stage described from the Philippines is *Neoperla obliqua* Banks, 1913 (Dela Cruz et al. 2018). Because of the very limited material and literature, the distinctive features of the nymphs are still unclear. In addition, when adults are collected, the females, which were not given proper taxonomic identification aside from its ootaxonomy, are also hard to identify. Describing the nymphs and female adults together with conspecific males using barcoding would somehow aid in overcoming the impediments to species identification in macroinvertebrate assessments. With the rise in popularity of eDNA technology in biodiversity surveys nowadays, the need of barcode references has never been more important than now (Balke et al. 2013; Lim et al. 2016; Fernández et al. 2018).

**Figure 8.**
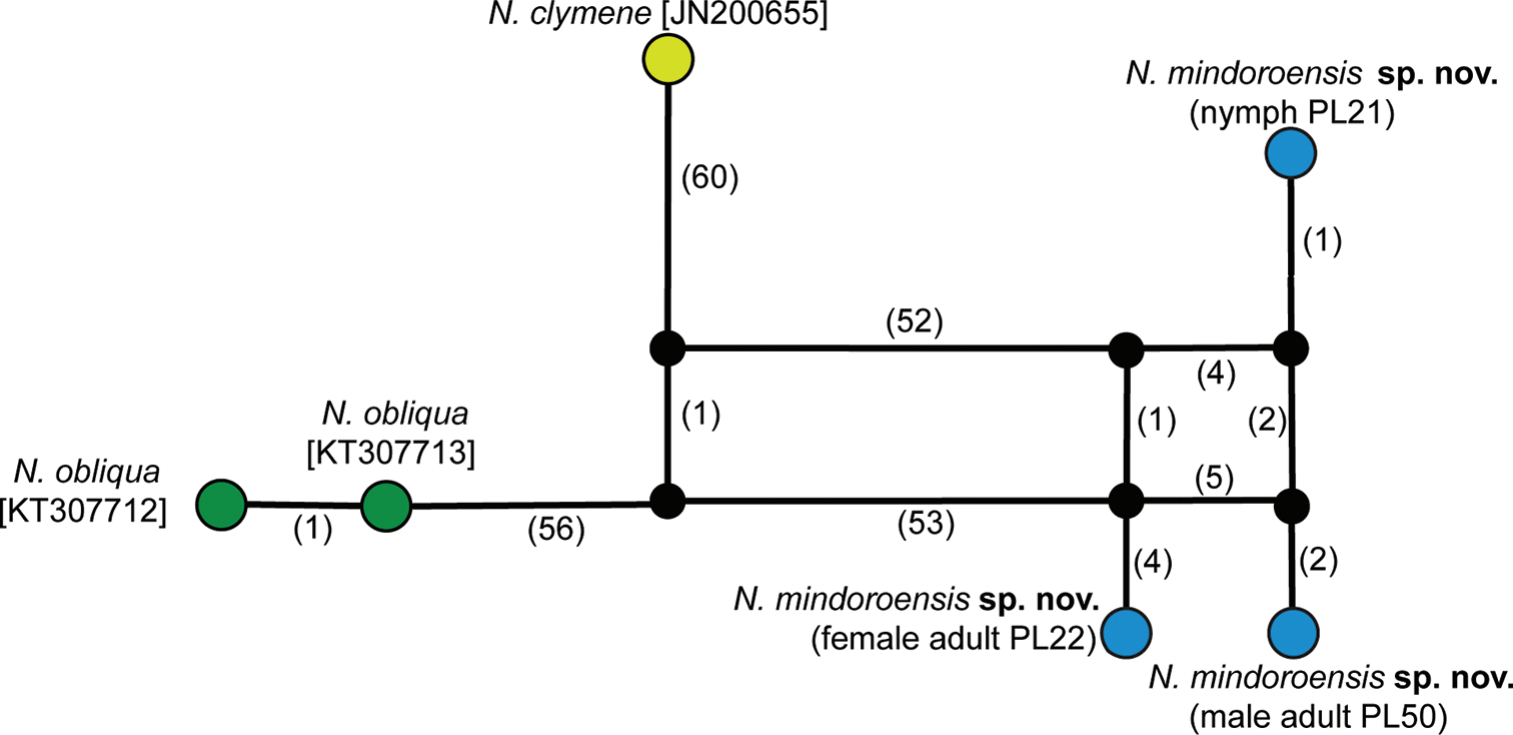
Statistical parsimony haplotype network of *Neoperla* samples and Genbank records from aligned sequences of 567 bp.

In this study, the 3.5% intraspecific divergence threshold was followed as observed in several EPT sequence divergence analyses (Zhou et al. 2010; Gattolliat et al. 2016). The maximum intraspecific divergence of 2.2% (Table 2) clearly contrasts from the minimum interspecific distance of 23.5%. The haplotype network association (Fig. 8) of the specimens does also support the morphological species concept. Barcoding of stonefly species in the Philippines has just started recently (Dela Cruz et al. 2016, 2018). Clearly, additional efforts must be done in collection and identification of stoneflies using integrative taxonomy to further advance the building of a comprehensive reference library which would aid studies on Plecoptera systematics and zoogeography.

**Table 2.** Intraspecific and interspecific pairwise distances of COI sequences based on Kimura-2-parameter (K2P) model.

		1	2	3	4	5	6
1	*N. mindoroensis* sp. nov. nymph PL21						
2	*N. mindoroensis* sp. nov. female adult PL22	0.022					
3	*N. mindoroensis* sp. nov. male adult PL50	0.009	0.020				
4	*N. obliqua* KT307712	0.243	0.237	0.245			
5	*N. obliqua* KT307713	0.245	0.235	0.243	0.002		
6	*N. clymene* JN200655	0.245	0.245	0.253	0.248	0.246	

*Neoperla mindoroensis* sp. nov. is a member of the *N. recta* Banks, 1913 species complex within the *N. montivaga* Zwick, 1977 species group (*sensu*Zwick 1983), which is recognized for its T7 and T8 with pointed processes, presence of basolateral lobes in the everted aedeagal sac, concentric and lateral folds visible around and in front of the receptacle attachment, and punctate, chorionic egg surface (Zwick 1983; Sivec 1984). With closest similarity to *N. nishidai* Sivec, 1984, this species complex now has six members, including *N. andreas* Sivec & Stark, 2011, *N. pseudorecta* Sivec, 1984, *N. recta* Banks, 1913, and *N. zwicki* Sivec, 1984 (Sivec and Stark 2011). The *N. recta* species complex has been proposed to have taken the “Formosa-Luzon migratory track” instead of the “Sumatra track” due to low species similarity with Borneo, Java, and Sumatra (Zwick 1986). The presence of *N. mindoroensis* sp. nov. on Mindoro Island, a large land mass between Luzon and Palawan, helps now to hypothesize how the species complex reached down to Palawan as well as to the oceanic islands of the eastern Philippines as some of its representatives have also been recorded in Cebu, Negros, Samar, and Mindanao (Sivec 1984; Sivec and Stark 2011).

The material treated here was exclusively retrieved from the Baroc River catchment as the main field research locality of the work group. During the long-term sampling program, the disturbed lower river reaches as well as various major and minor tributaries were accessed repeatedly. The new species was only found in rather undisturbed, clean tributaries (see Ecology), which suggests it has value as bioindicator for such habitats. However, to further assess its suitability and potential, an intensive ecological assessment is recommended.

## Supplementary Material

XML Treatment for Neoperla mindoroensis
